# SARS-CoV-2 vaccines–induced thrombotic thrombocytopenia: should we consider immuno-hypersensitivity?

**DOI:** 10.11606/s1518-8787.2021055003855

**Published:** 2021-10-18

**Authors:** Rine Christopher Reuben, Lillian Yami Adogo

**Affiliations:** I German Centre for Integrative Biodiversity Rersearch Germany German Centre for Integrative Biodiversity Rersearch (iDiv). Halle-Jena-Leipzig, Germany; II Bingham University Department of Biological Sciences Karu Nigeria Bingham University. Department of Biological Sciences. Karu, Nigeria

**Keywords:** COVID-19, prevention & control, SARS-CoV-2, immunology, COVID-19 Vaccines, Immunogenicity, Vaccine, Embolism and Thrombosis

## Abstract

The coronavirus disease 2019 (COVID-19) pandemic is significantly causing unprecedented clinical, socioeconomic, and public health challenges globally. The successful global administration of effective, safe and sustainable vaccine(s) is widely believed to be crucial in mitigating as well as preventing COVID-19. However, the rising cases of severe adverse events following immunization (AEFI) with COVID-19 vaccines including thrombosis, thrombocytopenia, and in some instances, death have created serious global concerns and could enormously contribute to vaccine hesitancy. Although the complete underlying pathophysiology and immunopathology of the COVID-19 vaccines related to AEFI, including thrombosis and/or anaphylaxis, are yet to be determined, exploring possible immuno-hypersensitivity could be crucial in the mechanisms associated with these reactions, thereby mitigating their occurrences as well as restoring confidence in vaccine administration for a COVID-19 free world.

## INTRODUCTION

Safe, secure, and viable vaccines are pivotal and sustainable to lessening and mitigating the devastating socioeconomic and health impacts of the current coronavirus disease 2019 (COVID-19) pandemic globally. Vaccines against the coronavirus (SARS-CoV-2) are the foremost critical countermeasure to fight the COVID-19 pandemic. By June 2021, at least 12 different COVID-19 vaccines were licensed or received approval for emergency use, and are currently administered in different countries: two RNA-based vaccines (Pfizer–BioNTech, and Moderna), four non-replicating adenovirus-vector based vaccines (Astra-Zeneca, Johnson & Johnson/Janssen, Gamaleya, and CanSino), four inactivated virus vaccines (Sinopharma, Sinovac, Sinopharma-Wuhan and Bharat Biotech) and two protein vaccines (Novavax and Vector Institute), respectively^1^.

### Adverse Events Following Immunization (AEFI) with COVID-19 Vaccines

Over one billion dosages of the COVID-19 vaccines have been administered across more than 100 countries according to the data collected by Bloomberg Vaccine Tracker. However, current reports of AEFI with COVID-19 vaccines have raised concerns and questions about their safety in humans^3,4^. Beginning in late February, 2021, cases of unusual thrombotic events in association with thrombocytopenia were observed in patients after vaccination with the ChAdOx1 nCoV-19, AstraZeneca vaccine, and the Johnson & Johnson/Janssen COVID-19 Vaccine^5^.

As of Tuesday, April 20^th^, 2021, the European Medicines Agency (EMA), a body in charge of the drug regulation in Europe, had reported more than 300 cases of rare blood clotting incidents along with low platelet counts worldwide after the use of COVID-19 vaccines. Based on the report, there were 287, 8, 25 and 5 cases of AEFI due to AstraZeneca, Johnson & Johnson’s, Pfizer and Moderna COVID-19 vaccines, respectively^8^. Similarly, 79 cases of rare blood clots with low platelets were also reported, along with 19 deaths in the UK out of over 20 million people who have been vaccinated with the AstraZeneca vaccine^9^. Initially, EMA and UK’s Medicines and Healthcare Regulatory Agency (MHRA), in reaction to the cases of rare blood clots and low blood platelets, stated the possibility of rare side effects due to the AstraZeneca vaccine, but could not, as of that time, establish a causal nexus^9,10^.

Also, the American Centers for Disease Control and Prevention (CDC) have reported cases of blood clots involving blood vessels in the abdomen, brain, and legs along with low platelet counts in some individuals who received Janssen COVID-19 Vaccine after about 1–2 weeks post-vaccination^7^. The clots became notable because some have occurred in unusual and deadly locations, such as in the veins that drain the brain (known as cerebral venous sinus thrombosis) and the abdomen (known as splanchnic vein thrombosis)^7^. We highlight that these reports came from developed countries with good disease reporting systems. Whereas millions of COVID-19 vaccine doses are concurrently administered in several developing countries, little has yet been reported about such AEFI and blood clotting. This could be attributed to their weak healthcare and disease reporting systems among other concerns.

### Presentation of Concerns

Although the administration of some COVID-19 vaccines, especially AstraZeneca, was briefly halted in several (European) countries due to severe AEFI^8,11,12^, the WHO and EMA stated that, despite the potential risks, the benefits of using the vaccine still outweigh the risks, hence, vaccination should continue^1,8^. These side effects have been observed to be more prevalent among young people than among older adults. Maheshi and colleagues^13^ reported a lower reactogenicity profile in older adults that received the ChAdOx1 nCoV-19 vaccine. Given this, some countries have announced that the administration of the vaccine in people aged 50 and above should continue until a new report is obtained^14,15^.

Apart from the different self-reported cases and/or media reports, recent studies have also reported AEFI with COVID-19 vaccines, including cases of thrombocytopenia and bleeding without thrombosis, as well as cases of thrombosis without thrombocytopenia reported among individuals who received RNA-based COVID-19 vaccines; Pfizer–BioNTech and Moderna, and also vaccine-induced thrombotic-thrombocytopenia (VITT) following the administration of adenovirus-vector based vaccines; Astra-Zeneca, J&J^16^. To understand the possible mechanisms involved in the development of thrombotic thrombocytopenia among patients who received the AstraZeneca vaccine, some researchers^17,18^recently reported severe and simultaneous blood clots and reduced platelets counts as immune-mediated response characterizing AstraZeneca vaccine-associated thrombotic thrombocytopenia.

Immunologically mediated hypersensitivity reactions following vaccine administration are not unusual, they are often uncomplicated, self-limiting, and sometimes not reproducible on re-exposure^19,20^. Althoughmost vaccines have the potential to trigger different immuno-hypersensitivity reactions^20^, complicated and severe post-vaccination immunologically-mediated hypersensitivity reactions, including blood clotting, are rare. Furthermore, the rising cases of COVID-19 vaccines AEFI globally could create serious concerns and enormously contribute to vaccine hesitancy worldwide.

Immune thrombocytopenia (ITP) often occurs when antibodies directly act against platelets. These autoantibodies attack platelets (including juvenile platelets) and usually clear them faster than in normal people, hence significantly reducing the platelet counts, and in more severe forms, bleeding symptoms. Over the years, rare cases of ITP have been associated with some infections, drugs, and vaccines^21^. In recent reports, almost all the patients that presented post-COVID-19 vaccines thrombosis had high levels of antibodies to platelet factor 4 (PF4)–polyanion complexes identified by ELISA as well as other platelet-based activation assays^24,25^. Therefore, since the constellation of post-COVID-19 vaccines thrombosis and thrombocytopenia, as revealed by clinical diagnoses, are yet to be fully understood, detailed mechanisms of immune system hyper reactions due to these vaccines would need to be explored extensively.

Whilst patients who reported thrombosis due to COVID-19 vaccines probably had no history of heparin therapy, proinflammatory cytokines such as interleukin-6 (IL-6), interleukin-1 (IL-1), and tumor necrosis factor-α (TNF-α) may be responsible for the spontaneous formation of platelet-activating anti-PF4/heparin antibodies similar to those formed in heparin-induced thrombocytopenia^26^. Proinflammatory cytokines are known to activate the coagulation system and further play a vital role in the down-regulation of important physiological anticoagulant pathways. These mechanisms have anti-inflammatory activities that cause up- and down-regulation of antithrombotic functions^27,28^. Toll-Like Receptors (TLRs) are a subclass of the pattern recognition receptor family that enhances the innate immune response against a wide range of molecules. TLRs are a major component of the innate immune system due to their capacity to effectively trigger inflammatory pathways and they are found on certain cells such as endothelial cells, platelets, and antigen-presenting cells^29^. The mechanisms by which the TLRs contribute to thrombosis are not fully understood. Notwithstanding, some studies on TLR2 and TLR4 in models of thrombosis have shown some possible association between TLRs, coagulation, and thrombosis^30,31^. Hence, the role of TLRs and their likely hypersensitivity reactions should be explored in the quest to decipher the cause of post-COVID-19 vaccination thrombosis.

It is crucial to realize that as current knowledge about corona virus biology evolves, our current understanding of SARS-CoV-2, COVID-19 pandemic, and various intervention measures including vaccine development and administration would keep advancing and changing as well. In order to halt the pandemic, science has been embroiled with uncertainties both in understanding COVID-19 spread and epidemiology, and also the outcomes of vaccine interventions at the individual and population levels. With all the uncertainties surrounding the COVID-19 vaccines, AEFI in individuals across age and gender, and the totality of the immune system involvement especially IgE in all the reported AEFI to the available mRNA COVID-19 vaccines is yet to be fully elucidated. In addition, the overall underlying pathophysiology and immunopathology of a case series or population-based study of the COVID-19 vaccine AEFI including thrombosis and/or anaphylaxis are yet to be determined and reported.

Diagnosing vaccine related AEFI need to be differential and encompassing, especially in a pandemic situation. With the increasing cases of AEFI due to COVID-19 vaccines, it is timely to consider immune-hypersensitivity and other immune-mediated phenomena, which should be given utmost priority. A better understanding of these AEFI, including thrombosis, along with the immune system and immunopathological continuum, would elucidate the mechanisms involved and further shed light on their control for optimum vaccine performance, hence curtailing the COVID-19 pandemic.

Furthermore, there are speculations that polyethylene glycol (PEG) , a constituent of COVID-19 vaccines, is a definitive allergen^32,33^. The atomic weight of PEG varies and PEG with higher molecular weight, when present in higher concentration, has been documented to promote allergic reactions^34^. However the extent and severity of these allergic reactions in humans are yet to be extensively studied.

### Conclusion and Future Direction

Our current understanding of the evolving events regarding the approach to COVID-19 vaccines production, administration, as well as the management of AEFI, can be applied to individual, community-based, national, regional, and global measures, including vaccine(s) deferral in efforts toward safe and sustainable mitigation of COVID-19 pandemic. Any approach generally accepted for COVID-19 control and prevention must balance benefits and risks, both in individual and population-based prevention, thereby mitigating risks for AEFI, including immune-hypersensitivity. Understanding these competing and crucial priorities remains a clinical and public health challenge. Nonetheless, with the rising cases of AEFI, including thrombosis and thrombocytopenia associated with COVID-19 vaccinations, it becomes imperative to consider mechanisms related to immune system hypersensitivity.

Furthermore, for individuals with COVID-19 vaccine-associated thrombosis as well as other AEFI, it is important to determine and identify various immunological and pathophysiological mechanisms of such reaction(s). Once immune-hypersensitivity is established among individuals with post-vaccination thrombosis, and they are required to receive additional vaccine dose(s), vaccine desensitization may be undertaken or, split doses of the vaccine may be administered especially in low-risk individuals^19^. Finally, apart from the active vaccine component (the antigen) which triggers an immune response, other sensitive vaccine constituents including adjuvants, residual protein, preservatives, acid stabilizers, salts, polyethylene glycol as well as other constituents which may induce immune-hypersensitivity should be evaluated in real-time immunopathological trials.
